# The Use of a Behavior Chain Interruption Strategy to Teach Mands for Help with an Adult with Intellectual Disability and Deaf-Blindness

**DOI:** 10.1007/s40616-024-00204-8

**Published:** 2024-02-21

**Authors:** Hannah E. Thompson, Robbie J. Hanson

**Affiliations:** Robbie Hanson, College of Education and Human Services, Lindenwood University, 209 South Kingshighway, St. Charles, MO 63301 USA

**Keywords:** Communication, Deaf-blind, Intellectual disability, Interrupted behavior chain, Mand training, Motivating operations

## Abstract

Individuals with deaf-blindness and co-occurring diagnoses, such as intellectual and developmental disabilities, may experience difficulty with independence, specifically with communication. One behavior-analytic procedure that may be useful for increasing independence and teaching communication to this population is the behavior-chain interruption strategy (BCIS). The current study examined the use of the BCIS to teach a 65-year-old deaf-blind participant with severe intellectual disability to use a SadoTech Elderly Monitoring Pager to notify others in the environment when help was needed. The researcher alternated between establishing operation (EO; help needed, items missing, or inoperable) and abolishing operation (AO; help not needed, items present, and operable) trials for three previously mastered daily living routines. The results demonstrated that following intervention, the participant used the device independently during EO trials and never used it during AO trials across behavior chains, and similar results were obtained during a treatment-extension phase. Limitations and implications for applied practice are discussed.

**Supplementary Information:**

The online version contains supplementary material available at 10.1007/s40616-024-00204-8.

The National Center on Deaf-Blindness (2023) reported that 10,441 children and approximately 40,000 adults had a diagnosis of deaf-blindness in the United States in 2021, and roughly 90% of these individuals have a co-occurring medical, physical, or cognitive diagnosis (National Library Service for the Blind and Print Disabled, 2023). Individuals with deaf-blindness experience a range of vision and hearing loss which may impact independence, such as daily living and communication. One behavior-analytic procedure that may be useful for increasing independence and teaching communication to this population is the behavior-chain interruption strategy (BCIS).

The BCIS typically involves prearranging a disruption during a specific step of a previously mastered routine to contrive an establishing operation (EO) for manding. Mands can include requests for missing items (e.g., “Can I have the [item]?”), information (e.g., “Where is it?”), or non-specific mands such as help (e.g., “Help me,” Ban & McGill, 2023; Carnett et al., 2017). Interrupting a step in the chain can be conceptualized as using transitive conditioned motivating operations (CMO-T) to increase (or decrease) the value of another stimulus in the environment and increase (or decrease) behavior. For example, when an individual reaches the step during hand washing, in which the item necessary to complete the step is missing or inoperable (the soap is missing, the faucet knob cannot be turned), this may serve as a CMO-T making the retrieval of the missing item or the repair of the broken item valuable. This then may increase mands for the missing item, information leading to the location, or for help. Cengher et al. (2022) conducted a literature review on mands for information and results showed MOs were manipulated in a variety of ways (e.g., hiding an object, preventing completion of activity, etc.) and across autoclitic frames (e.g., “who,” “how,” etc.). Cengher et al. found that most studies included only an EO condition, which limits conclusions about the function of the response, but there was an increasing trend of rotating EO and AO conditions in the literature. The authors recommended future research examine teaching mands for information with other populations, as most participants were under the age of 18, had an autism diagnosis, communicated vocally, and none included deaf-blind individuals.

Although some previous studies have taught communication responses to deaf-blind individuals using behavioral procedures (e.g., differential reinforcement, shaping) and adaptive equipment (e.g., microswitches, textures, line-drawings), most have focused on teaching children and none have used an interrupted chain to teach mands to deaf-blind adults who are severely impacted (Durand & Kishi, 1987; Parker et al., 2007; 2008; Romer & Schoenberg, 1991; Sigafoos et al., 2008). Thus, additional research on teaching communication and manding to the deaf-blind adult population is warranted. The purpose of the current study was to examine the use of the BCIS to teach an elderly individual with bilateral deaf-blindness and severe intellectual disability to mand for help during previously mastered routines. A rotating control (AO) condition when help was not needed and a treatment extension phase to assess responding to untrained EOs were included.

## Method

### Participant and Setting

A 65-year-old, Caucasian male diagnosed with severe intellectual disability and bilateral deaf-blindness was included. Due to the level of cognitive impact, he was legally conserved by a family member who provided written consent. The participant provided assent prior to and throughout each session by remaining seated, standing in the immediate area, and/or remaining engaged with the researcher in the absence of maladaptive behavior. If the participant engaged in maladaptive or precursor behavior (e.g., clapping hands together firmly), sessions were terminated by the researcher prompting the “all done” sign by tapping on the participant’s elbows. The participant and his conservator did not receive compensation for participation, and the researcher informed the participant’s conservator that participation could be terminated at any time. The researcher conducted the study within the adult residential facility (ARF) where the participant resided with 24-h care from a licensed vocational nurse (LVN) and direct support professionals (DSPs). All aspects of the study were approved by the university’s institutional review board.

The participant was reported to work as an electronic parts handler from 1989–2005 in a vocational group in which he communicated using unspecified tactile prompts and adapted American Sign Language (ASL; signing into the palm of his hand). The participant resided in a developmental center from 2005–2017 prior to moving into his current residence. No additional documentation or skills assessments were made available to the researcher.

At the time of the study, the participant independently used the restroom, manipulated the bidet, dressed himself, fed himself, and navigated his home with tactile cues (i.e., rectangular/circular wooden plaques) mounted to the walls. The participant was reported to meet mastery criterion (i.e., 80% accuracy on average for 3 months) for the adapted ASL sign “more” but all other ASL targets (i.e., adaptive signs for “choices,” “all done,” and “eat”) required prompting from others. Per staff report, the participant engaged in self-injurious behavior (SIB; biting his hand) on average three times per month when he required assistance or when basic needs were not met (e.g., hunger, thirst, etc.).

### Materials

The researcher selected a SadoTech Elderly Monitoring Pager (referred to as “device” hereafter) supplied with a monitoring pager worn on a lanyard and a luminating receiver for the participant to request help (see Appendix A). When the monitoring pager’s button was pressed, the receiver illuminated and emitted an auditory signal. Additionally, the researcher carried over a Sensory Slap Fidget Bracelet (see Appendix A) from the participant’s regular programming to indicate that the researcher was present. Prior to beginning each session, the researcher placed the bracelet on her right wrist, approached the participant, and placed her hand near the participant’s hand so that they could touch the bracelet.

### Dependent Variables and Response Definitions

The dependent variable was the percentage of independent correct responding for device usage. Correct responding was defined as independently reaching for and manipulating the device with one or both hands and pressing the button using one or more fingers when help was needed. The researcher used touch cues (i.e., tapping the participant’s elbow twice and waiting 1 s), partial-physical (i.e., wrapping palm around elbow and providing slight pressure while pushing forward toward the device), and full-physical (guiding with hand-over-hand assistance to use the device) prompts to teach the response. Incorrect responses on EO trials (i.e., when help was needed) were defined as holding the device without pressing the button or engaging in unrelated or maladaptive behavior; an incorrect response on AO trials (i.e., when help was not needed) was using the device.

### Procedure

Prior to beginning each session, the researcher placed the tactile bracelet on her right wrist, approached the participant, and placed her hand near the participant’s hand. If the participant did not move away or engage in maladaptive behavior, the researcher began the session. Sessions included both EO (i.e., help was needed to complete the routine) and AO (i.e., help was not needed to complete the routine) trials programmed during previously mastered behavior chains (i.e., toileting, meals, and dressing; see Table 1 and Online Supporting Information for details regarding manipulated chains) at times when they were normally completed (e.g., when the participant needed to use the bidet, after dinner was prepared, and before bed). The researcher remained the same distance from the participant on all trials across phases.

**Table 1:** Intervention scenarios during EO trials

Scenario	Step
Bathroom	LVN switched bidet off prior to accompanying participant to restroom.
	When participant finished void, he triggered bidet (turned dial but no water flowing for cleanliness).
	After 1 s, participant prompted to use device and help provided (LVN touched participant’s shoulder with light pressure and switched the bidet on).
	Researcher instructed LVN to guide the participant’s hand to bidet to turn dial to finish bathroom routine.
Mealtime	Researcher instructed LVN to provide participant with his meal without placing utensils next to his bowl.
	Participant felt the areas surrounding his bowl and researcher allowed 1 s to elapse prior to prompting device use and providing help (LVN touched participant’s shoulder and provided utensils).
Dressing	LVN instructed to remain in doorway as the participant was getting dressed in his room.
	As participant got dressed the LVN was instructed to inform the researcher when the participant began to search for a shirt.
	When the participant began searching his dresser drawers for shirts, he was allowed to touch the entirety of the empty drawer.
	After the participant felt inside the empty drawer, researcher allowed 1 s to pass prior to prompting device use and providing help (LVN touched participant’s shoulder and granted access to laundry basket with the participant’s shirts inside). The participant then chose the desired shirt at his leisure.

LVN = licensed vocational nurse. See Online Supporting Information for full descriptions of scenarios

#### Establishing Operation (EO) Trials

On EO trials, the researcher arranged for materials for one step in the chain to be broken, inoperable, or missing. For example, the bidet was made inoperable by switching it to the off position, the utensils were missing when a meal was served, and shirts were missing from the dresser drawer.

#### Abolishing Operation (AO) Trials

On AO trials, the researcher did not arrange for any materials to be broken, inoperable, or missing.

#### Baseline

On EO trials in baseline, the researcher waited 15 s once the participant reached the contrived step. If the participant used the device independently, reinforcement in the form of help (i.e., providing the missing item or fixing the broken object) was provided. Following 15 s without the participant using the device, the researcher ended the trial and restored the environment (e.g., switched bidet to on position, provided utensils, placed shirts in dresser). During AO trials, the researcher used the least intrusive prompt for the participant to continue to the next step of the routine if they used the device. If the participant did not use the device during AO trials, the participant could complete the routine without interruption.

#### Intervention

In the intervention phase, the participant completed trials for a maximum of 10 min up to three times per day (e.g., missing shirts during dressing routine was one trial which could have lasted one full 10 min period due to the duration of the dressing routine itself). The researcher began by providing the participant with errorless prompting in which the researcher contrived the same three situations (EO trials) from baseline and used most-to-least prompting. Errorless prompting was discontinued after completing trials for a total of 20 min. After 20 min, we advanced to a progressive-prompt delay (i.e., 1 s, 2 s, and 3 s delay) wherein the delay was lengthened following 10 trials without prompt resistance. Across intervention, the participant was exposed to each scenario simultaneously while rotating AO/EO trials. The researcher alternated AO trials in which identical scenarios were presented without researcher manipulation (with the researcher the same distance away). Once the participant reached 80% correct responding across all scenarios, we began the treatment extension.

#### Treatment Extension

After reaching the mastery criterion, the researcher manipulated a different step in each of the three routines (see Table 2 and Online Supporting Information for detailed scenario information) and alternated between EO and AO trials. The researcher used least-to-most prompting if the participant did not independently use the device.

**Table 2:** Treatment extension scenarios during EO trials

Scenario	Step
Bathroom	Researcher instructed DSPs to tape down the toilet flushing handle to make it inoperable (unable to flush the toilet).
	DSPs instructed to observe for when the participant began to push the handle down to inform the researcher.
	The researcher allowed for 10 s to elapse prior to recording responses or prompting device use if needed and provided help.
Mealtime	As the participant was out of his recliner (e.g., while eating a meal), the researcher placed three boxes of printer paper into the seat of the recliner.
	As the participant returned to his recliner, researcher allowed participant to feel what was on his chair and 10 s elapsed prior to recording the response or prompting device use if needed and provided help.
Dressing	The researcher rigged the participant’s closet door shut (could not be opened).
	The researcher allowed the participant 10 s prior to recording his response or prompting device use if needed and provided help.

DSPs = direct support professionals. See Online Supporting Information for full descriptions of scenarios

## Experimental Design

A two-phase (baseline and intervention) multielement design was used in which the researcher alternated EO and AO trials; intervention was implemented with all routines simultaneously following one baseline session each. We did not conduct multiple baseline sessions with each routine because staff reported the participant did not communicate independently for help and had a history of engaging in SIB when assistance was needed; therefore, timely introduction of the intervention was important for the participant and staff.

### Interobserver Agreement and Treatment Integrity

Interobserver-agreement data were collected by a secondary observer (ARF’s LVN) across 100% of trials across phases. We calculated interobserver agreement by dividing the total number of agreements by the total number of agreements and disagreements and multiplying by 100; agreement averaged 93% (range, 90%-100%). The ARF’s LVN collected treatment integrity data during all trials in the intervention phase. We calculated treatment integrity by dividing the number of correctly implemented trials by the total number of trials and multiplying by 100; integrity averaged 95% (range, 95–100%).

## Results

**Fig. 1:**
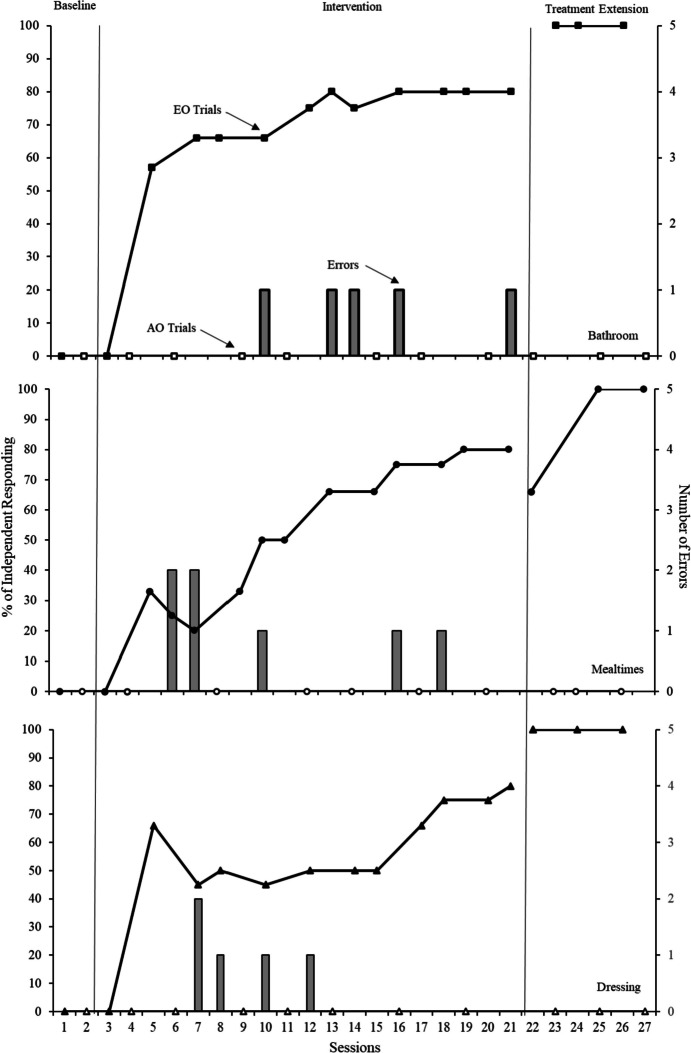
Percentage of independence for device usage and number of errors across scenarios. *Note*. Errorless prompting began on session 3 for all routines, a 1 s time delay began during session four, a 2 s delay began during session seven (bathroom/mealtimes) and session eight (dressing). The final time delay (3 s) began during session 10 (mealtimes), session 12 (dressing) and session 13 (bathroom)

Figure 1 shows the results across baseline, intervention, and treatment extension. During baseline, the participant did not use the device on EO or AO trials across any presented scenarios. When intervention was introduced, use of the device during EO trials increased and independent responding averaged 74% (range, 57–80%) for the bathroom scenario, 56% (range, 25–80%) for the mealtime scenario, and 61% (range, 45–80%) for the dressing scenario. Throughout intervention, the participant never used the device during AO trials for any of the presented scenarios. On EO trials in the treatment extension phase, the participant utilized the device with 100% independence for the bathroom and dressing scenarios, and averaged 88% independence (range, 66–100%; one touch cue prompt was provided following an incorrect response) for the mealtime scenario. The participant did not use the device during any AO trials during treatment extension. Incorrect responses (errors) consisted of attempting to continue with the routine without utilizing the device when help was needed during EO trials and ranged from 1–2 errors when the time delay was increased.

## Discussion

The current study employed the BCIS to teach a 65-year-old male with severe intellectual disability and deaf-blindness to mand for help using a device when steps were disrupted during previously mastered routines. Results showed the participant used the device independently during the intervention and treatment extension on EO trials and never used the device during AO trials. The inclusion of both EO and AO trials in the multielement design increased the likelihood that the use of the device was under mand control. To date, research is extremely limited for adults with intellectual disability and deaf-blindness, and the current study demonstrates that using an interrupted-chain procedure could be beneficial when teaching mands to these individuals.

This case study had several limitations. First, external validity is limited. We included only one elderly participant, and it is uncertain whether similar results would be obtained with other participants with similar needs. Second, our experimental design limits conclusions about functional relationships. We conducted just one baseline session per scenario and did not assess responding with the manipulations used in the treatment-extension phase prior to intervention. Baseline data were limited because of the participant’s history of engaging in SIB when he required assistance and because prior to the study, he did not communicate independently to request help. Additionally, we did not stagger the introduction of the intervention across scenarios, did not withdraw treatment, and did not measure SIB along with mands for help. Stronger control would be demonstrated by conducting additional baseline sessions across all routines, including those used to assess for generalization (Miguel, 2017), and staggering the introduction of intervention across scenarios. Anecdotally, the researcher did not observe any SIB throughout the study, and the ARF’s staff did not record any instances outside of sessions. Nevertheless, future research should collect data on maladaptive behavior to determine the influence of the intervention. Third, treatment integrity data were collected during intervention only and future research should collect procedural fidelity data across all phases.

Despite limitations, the results of this study are encouraging and contribute to our understanding of effective interventions for individuals with intellectual disabilities and multi-sensory impairments like deaf-blindness. Future research could consider assessing the function of device usage as it is possible that the participant used the device to obtain help, gain attention from the researcher, or avoid prompting. Future research should examine this by including trials in which using the device results in attention but without help. Additionally, future studies could consider assessing participant preference for communication options, collecting data on device usage over time, and assessing social validity of the intervention procedure and device.

### Electronic supplementary material

Below is the link to the electronic supplementary material.

Supplementary file1 (DOCX 18 KB)

## Data Availability

All data are available upon request.

## Appendix A

SadoTech Elderly Monitoring Pager

Sensory Slap Fidget Bracelet Bands (2-Pack)
