# Supplementary material for: The Use of a Behavior Chain Interruption Strategy to Teach Mands for Help with an Adult with Intellectual Disability and Deaf-Blindness
Source: Anal Verbal Behav. 2024 Feb 21;40(1):88–98. doi: 10.1007/s40616-024-00204-8 (PMC11217214; doi:10.1007/s40616-024-00204-8)
Supplement: Supplementary file 1 — Supplementary file1 (DOCX 18 KB) [file 40616_2024_204_MOESM1_ESM.docx]

**The Use of a Behavior Chain Interruption Strategy to Teach Mands for Help for an Adult with Intellectual Disability and Deaf-Blindness**

**Supplemental Information**

**Baseline and Intervention Scenario Details**

During all scenarios the researcher utilized the LVN as a secondary person to assist. During the first scenario (bathroom routine), the researcher instructed the LVN to switch off the bidet prior to accompanying the participant to the restroom. While standing outside the restroom, the researcher instructed the LVN to signal when the participant was finished with either urinating or a bowel movement. When the void was completed, the researcher allowed the participant to trigger the bidet. After the participant turned the dial (with no water flowing for cleanliness), during intervention the researcher allowed for 1 s to elapse prior to prompting the desired response. This was to ensure that the participant experienced the contingency of the bidet’s water not flowing (unable to successfully clean intimate areas prior to getting off toilet seat). Once the device’s button was pushed, the participant was provided with assistance. In order for the participant to be notified that someone was there to assist, the researcher instructed the LVN to touch the participant’s shoulder with light pressure and switch the bidet to “on.” The researcher then instructed the LVN to guide the participant’s hand to the bidet turns dial. The participant then successfully completed his bathroom routine.

In the second scenario (mealtimes), the researcher instructed the LVN to provide the participant with his meal without placing utensils next to his bowl. The participant was allowed to search the areas surrounding his bowl of food. After the participant felt around the sides of the bowl, the researcher allowed for 1 s to elapse prior to prompting during intervention. When the device was successfully used, the LVN utilized the tactile signal (shoulder touch with light pressure) to notify the participant that help was present. After the signal was provided, the LVN was instructed to deliver the required utensils by placing them in the participant’s hand. The participant was able to eat the entirety of his meal without interruption.

Within the final scenario (dressing routine), the LVN was instructed to remain in the doorway as the participant was getting dressed in his room. As the participant successfully got dressed the LVN was instructed to inform the researcher when the participant began to search for a shirt. When the participant began searching his dresser drawers for shirts, he was allowed to touch the entirety of the empty drawer. After the participant felt inside the empty drawer, the researcher allowed 1 s during intervention to pass prior to prompting the participant to utilize the device. When the device’s button was pressed, the LVN provided the tactile signal and assisted the client. The LVN was instructed to grant access to the laundry basket with the participant’s shirts inside. The participant then chose the desired shirt at his leisure.

**Treatment Extension Scenario Details**

For the first probe, the researcher instructed the DSPs to tape down the toilet flushing handle to make it inoperable (unable to flush the toilet). The DSPs were instructed to observe for when the participant began to push the handle down and inform the researcher. The researcher allowed for 10 s to elapse prior to recording responses or prompting if needed. Within the second probe the researcher rigged the participant’s closet door shut. As the participant was getting ready for the day the researcher observed the participant navigate towards the closet. The researcher allowed the participant 10 s prior to recording his response or prompting if needed. The final probe consisted of placing items in the participant’s recliner that needed to be removed. As the participant was out of his recliner (e.g., while eating a meal), the researcher placed three boxes of printer paper into the seat of the recliner. As the participant returned to his recliner, the researcher allowed the participant to feel what was on his chair and 10 s elapsed prior to recording the response or prompting if needed. The participant was provided with reinforcement (receiving help as needed) for correct responses during probes or was provided prompts to emit a correct response (using least-to-most prompting) for incorrect responses.
